# Supplementary material for: Early Rehabilitation Exercise after Stroke Improves Neurological Recovery through Enhancing Angiogenesis in Patients and Cerebral Ischemia Rat Model
Source: Int J Mol Sci. 2022 Sep 10;23(18):10508. doi: 10.3390/ijms231810508 (PMC9499642; doi:10.3390/ijms231810508)
Supplement: Supplementary file 1 [file ijms-23-10508-s001.zip › ijms-1888105-supplementary.pdf]

Supplementary material

Table S1 General statistics of demographic data of acute ischemic stroke patients（n=75，%）

Item		Number （n）	Composition ratio （%）
Gender	Male	47	62.7
	Female	28	37.3
Marital status	Single	0	0
	Married	69	92
	Divorced	3	4
	Widowed	3	4
Educational level	Llliteracy	20	26.7
	Elementary school	23	30.7
	Junior high school	18	24
	High School	5	6.7
	College and above	9	12
Occupation	Work	5	6.7
	Retire	14	18.7
	No job or layoff	56	74.7
Household per capita monthly income *(in yuan)*	< 3000	49	65.3
	3000-5000	20	26.7
	> 5000	6	8
Caregiver	Relatives	65	86.7
	Professional carer or nanny	8	10.7
	No	2	2.7
Smoking	Yes	18	24
	No	57	76
Drinking	Yes	28	37.3
	No	47	62.7
Hypertension	Yes	31	41.3
	No	44	58.7
Diabetes	Yes	26	34.7
	No	49	65.3
Coronary heart disease	Yes	18	24
	No	57	76
Cerebral infarction hemisphere	Left	36	48
	Right	39	52
